# Eumycetoma on the Back

**DOI:** 10.4269/ajtmh.24-0699

**Published:** 2025-02-18

**Authors:** Mamadou Ball, Dallas J. Smith

**Affiliations:** ^1^ Department of Dermatology, National Hospital Center, Nouakchott, Mauritania;; ^2^ Mycotic Diseases Branch, Centers for Disease Control and Prevention, Atlanta, Georgia

**Figure 1.**
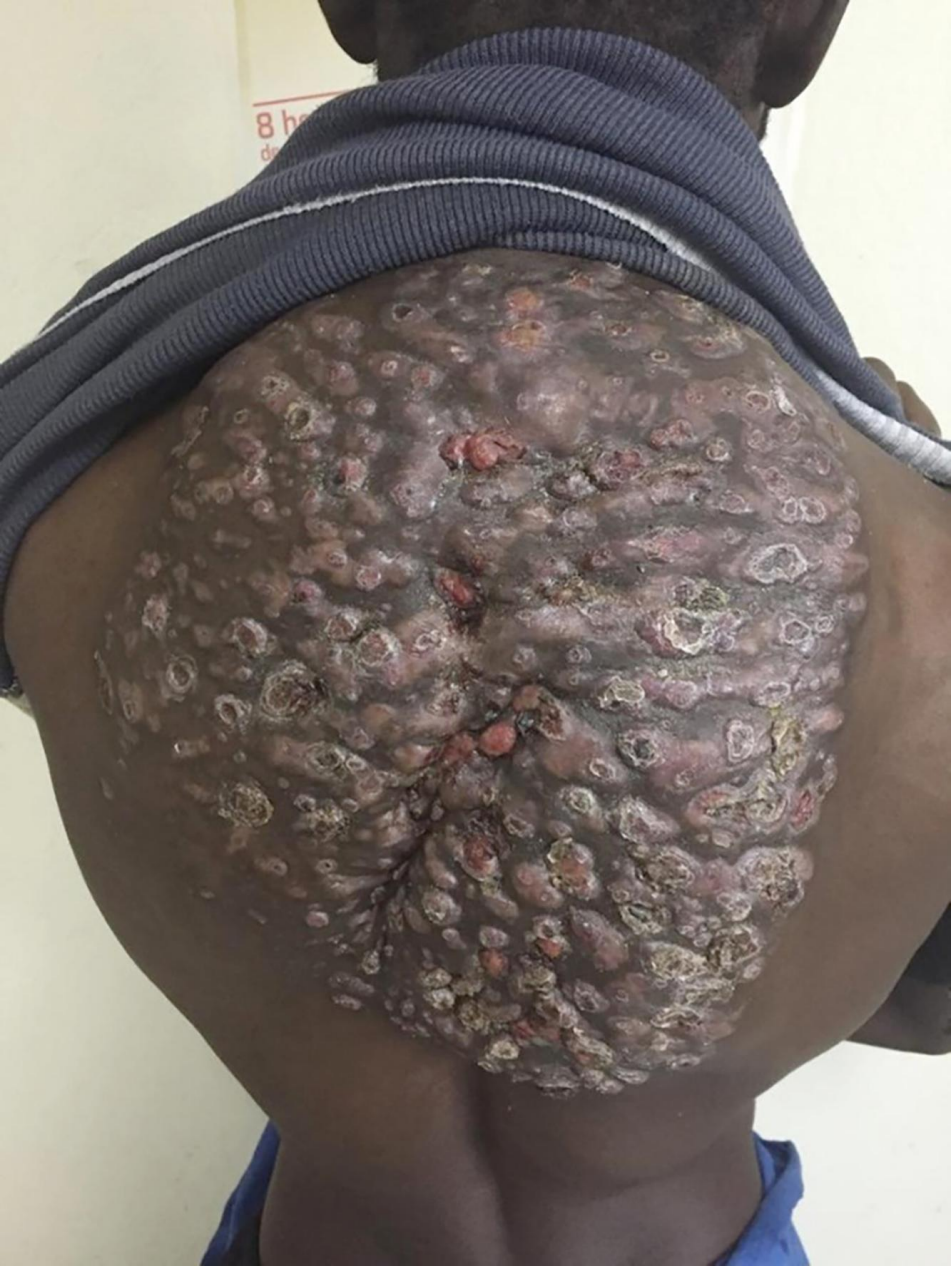
Eumycetoma on the back.

A healthy 35-year-old man from Mauritania presented to the dermatology department with a several-year history of evolving lesions on his back (Figure 1). The patient, a rural shepherd without local access to health care, did not recall any specific injury at the infection site. A physical examination was notable for a painless subcutaneous mass, multiple sinuses, and discharge containing grains. No systemic symptoms were identified. Material from a back lesion was obtained for microbiological studies. Black grains were visualized by using direct microscopy. A diagnosis of eumycetoma (mycetoma caused by fungi) was made.^1^ Supplies for performing a fungal grain culture, a punch biopsy, histopathology, and molecular diagnostics were unavailable; therefore, the genus and species could not be identified. Antifungals, including itraconazole, were not available through the public health care system, and the patient was unable to afford antifungals from a private pharmacy. The patient returned to his home in rural Mauritania and was lost to follow-up.
